# Performance of PEN‐FAST and CEPH‐FAST for Cephalosporin Allergy Delabeling

**DOI:** 10.1002/clt2.70178

**Published:** 2026-05-27

**Authors:** Deniz Göcebe, Hannah Nürnberg, Alexander Enk, Elham Khatamzas, Knut Schäkel

**Affiliations:** ^1^ Department of Dermatology University Hospital Heidelberg Heidelberg Germany; ^2^ Department of Cardiology, Angiology and Pneumology University Hospital Heidelberg Heidelberg Germany; ^3^ Hospital Pharmacy University Hospital Heidelberg Heidelberg Germany; ^4^ Department of Infectious Diseases and Tropical Medicine Center for Infectious Diseases University Hospital Heidelberg Heidelberg Germany; ^5^ German Center for Infection Research (DZIF) Partner Site Heidelberg University Hospital Heidelberg Germany

**Keywords:** antibiotic allergy, CEPH‐FAST, cephalosporin allergy, drug allergy, PEN‐FAST

## Abstract

**Background:**

Unverified antibiotic allergy labels lead to suboptimal antimicrobial prescribing and increased antibiotic resistance. Cephalosporins are among the most frequently administered antibiotics and cephalosporin allergy labels increasingly limit optimal therapy. Clinical decision rules that enable delabeling of low‐risk patients by non‐allergists are emerging as important tools. The PEN‐FAST score is a validated rule for identifying low‐risk penicillin allergy labels suitable for direct drug challenge. CEPH‐FAST is a recently proposed adaptation for cephalosporin allergy. We evaluated the diagnostic performance of PEN‐FAST and CEPH‐FAST for cephalosporin allergy.

**Methods:**

In this single‐center retrospective cohort study, 100 adults with reported cephalosporin allergy labels underwent allergy assessment including skin testing and drug provocation testing at a tertiary allergy clinic in Heidelberg, Germany. Logistic regression was used to identify predictors of confirmed allergy. Diagnostic performance of PEN‐FAST and CEPH‐FAST scores was assessed using sensitivity, specificity, predictive values, and area under the receiver operating curve (AU‐ROC).

**Results:**

Cephalosporin allergy was confirmed in 48 of 100 patients. PEN‐FAST categorized 20 patients at low risk, of whom one had a positive skin test (NPV 95.0%, sensitivity 97.9%, AU‐ROC 0.769). CEPH‐FAST classified 42 patients low‐risk, but 12 had confirmed allergies (NPV 71.4%, sensitivity 75.0%, AU‐ROC 0.667). Higher PEN‐FAST and CEPH‐FAST scores were significantly associated with confirmed allergy.

**Conclusion:**

PEN‐FAST demonstrated high sensitivity and negative predictive value for identifying low‐risk cephalosporin allergy and may support safe delabeling strategies. In contrast, CEPH‐FAST showed reduced diagnostic performance in this cohort, highlighting the need for further validation before routine clinical implementation.

## Graphical abstract

Evaluation of PEN‐FAST and CEPH‐FAST in 100 patients with cephalosporin allergy labels showed that PEN‐FAST reliably identified low‐risk patients suitable for direct drug challenge, while CEPH‐FAST demonstrated reduced diagnostic performance.

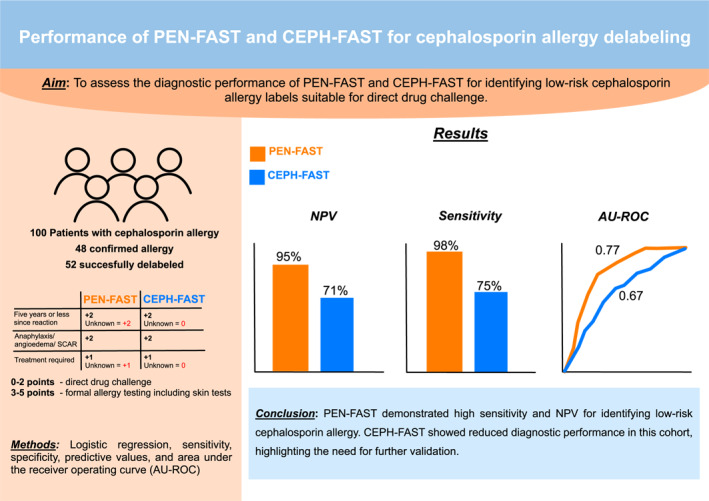

**Abbreviations**
AU‐ROC: area under the receiver operating curve
CI: confidence interval
DPT: drug provocation test
ICU: intensive care unit
IDT: intradermal testing
NPV: negative predictive value
PPV: positive predictive value
SCAR: severe cutaneous adverse reaction

## 1 Introduction

Beta‐lactam allergy labels affect approximately 5%–15% of patients, restricting the use of first‐line antibiotics in clinical practice [1, 2]. This results in increased use of broad‐spectrum alternatives, higher rates of antimicrobial resistance and surgical site infections, longer hospital stays, and elevated healthcare costs [3]. Cephalosporins are among the most frequently prescribed antibiotics in both Europe and the United States, in the outpatient as well as inpatient setting including peri‐operative antibiotic prophylaxis [4]. Cephalosporin allergy labels are increasingly recognized as a significant barrier to optimal antibiotic use [5].

Unlike penicillin, cephalosporin skin testing is not well standardized or fully validated, and its diagnostic performance remains controversial [5]. Although skin testing remains useful particularly in immediate allergies [6], false‐positive skin test results for cephalosporins have been reported [7], making clinical history‐based approaches essential for delabeling patients with low‐risk histories.

In recent years, substantial progress has been made to enable antibiotic allergy delabeling by non‐allergists [8, 9]. Direct drug challenges, performed without prior skin testing, are well supported by evidence as a safe and efficient method for penicillin allergy delabeling [10, 11]. Similar evidence is now emerging for cephalosporin allergy delabeling [12, 13]. Structured risk stratification using the PEN‐FAST score allows faster removal of incorrect labels by selectively enabling direct drug challenges in low‐risk patients carrying a penicillin allergy label [10, 14, 15, 16, 17, 18, 19, 20, 21, 22, 23]. In addition to its high negative predictive value (NPV), the safety of direct challenges using PEN‐FAST for penicillin allergy delabeling demonstrated to be non‐inferior compared to formal allergy testing using skin tests [15]. PEN‐FAST has proven applicable not only to penicillin but also to sulfonamides [24, 25].

Despite these advances, correcting cephalosporin allergy labels remains a critical opportunity to further improve antimicrobial stewardship and patient outcomes. Most delabeling approaches have focused on penicillin, and cephalosporin‐specific decision tools remain understudied. Recent evidence indicates that cephalosporin allergy labels can also be safely removed using a structured delabeling protocol based on PEN‐FAST which adopts an identical approach to identify patients eligible for direct drug challenge [26]. This newly developed CEPH‐FAST score incorporates only minor modifications in the timing and treatment categories of the original PEN‐FAST scoring system and has demonstrated a comparable NPV [26]. Unlike PEN‐FAST, the CEPH‐FAST score is more permissive, as it does not assign points when the timing of the reaction or whether treatment was received is unknown. This distinction makes CEPH‐FAST potentially more applicable in clinical practice, where patients often cannot recall precise details of their reaction history.

In this study, we conducted a retrospective analysis of patients with a cephalosporin allergy label evaluated in a specialized allergy clinic of a German hospital to assess predictors of allergy outcomes. We examined the recently published CEPH‐FAST score and compared its performance with the original PEN‐FAST score in identifying patients eligible for direct drug challenges. The aim was to evaluate whether the more permissive CEPH‐FAST would classify a greater proportion of patients as low risk while maintaining the high negative predictive value and safety profile observed with PEN‐FAST.

## 2 Methods

### 2.1 Ethics

The study was performed according to the Declaration of Helsinki and approved by the local ethics committee of the medical faculty of the University of Heidelberg, Germany (S‐004/2025). Data collection did not require informed consent. Data were obtained after approval of the ethics committee.

### 2.2 Study Design and Setting

This was a single‐center retrospective study of adult outpatients with a documented cephalosporin allergy label who underwent allergy evaluation between January 2004 and December 2024 at the tertiary referral allergy clinic of the Department of Dermatology, University Hospital Heidelberg, Germany. Physicians extracted and reviewed the datasets, which included detailed clinical history obtained directly from patients, routine information on patient characteristics, and allergy testing outcomes. The study was reported in accordance with the STARD 2015 guidelines [27].

### 2.3 Eligibility Criteria

Adult patients (≥ 18 years) with a reported cephalosporin allergy label who underwent allergy testing with the implicated cephalosporin were eligible for inclusion. Patients underwent any combination of skin prick testing, patch testing, and, if negative, consecutive drug provocation testing (DPT).

Patients were excluded if allergy testing was declined or discontinued, if DPT was performed with an antibiotic other than the implicated cephalosporin, or if the allergy assessment was incomplete. When patients were unable to reliably identify the implicated cephalosporin, the suspected agent was determined based on referral documentation and available medical records. Patients in whom the culprit drug could not be reasonably identified, or who underwent testing with an alternative beta‐lactam selected based on clinical indication, were excluded from the final analysis. Detailed inclusion and exclusion criteria are provided in the Supporting Information S1.

### 2.4 Allergy Assessment and Outcome Definitions

Skin tests were performed in accordance with EAACI recommendations [28]. The allergy label was confirmed if any of the above‐mentioned tests showed a positive result in accordance with local diagnostic protocols (detailed information is reported in the Supporting Information S1). Delabeling was considered achieved only when a negative DPT with the implicated cephalosporin was performed. Intradermal testing (IDT) was not performed, as it is not included in the hospital's standard diagnostic protocol for cephalosporin allergy. Drug provocation tests were performed in an inpatient or outpatient setting with a 2‐step challenge of a full therapeutic dose (detailed information is reported in the Supporting Information S1).

### 2.5 Cephalosporin‐Specific IgE Measurement

For patients with a cefaclor allergy label additionally measurement of cephalosporin‐specific IgE was available. Measurements were conducted using the ImmunoCAP system (Thermo Fisher Scientific) at the accredited laboratory of the University Hospital Heidelberg. Cefaclor‐specific IgE levels ≥ 0.35 kU/L were interpreted as positive and the cefaclor allergy label was confirmed if the patient's reported reaction was consistent with an immediate‐type allergic response without proceeding to DPT according to local protocol.

### 2.6 PEN‐FAST and CEPH‐FAST Scores

The three PEN‐FAST/CEPH‐FAST criteria were assessed in all patients. These include: two points for a reaction occurring within the past 5 years, two points for the presence of anaphylaxis, angioedema, or severe cutaneous adverse reactions (SCARs), and one point if systemic treatment was required (detailed information on the definitions of anaphylaxis and SCAR is reported in the Supporting Information S1) [14, 26].

The key distinction between the two scoring systems lies in the handling of incomplete historical information (Supporting Information S1: Table S1). The PEN‐FAST rule assigns two points for the timing of the allergic reaction and one point for the treatment category if the details are unknown [14]. In contrast, CEPH‐FAST does not assign any points when information on reaction timing or treatment is unknown, rendering it more permissive in patients with incomplete allergy histories [26].

For both decision rules, a cutoff score of 2 points is used and patients scoring 0–2 points are considered eligible for direct drug challenges.

### 2.7 Statistical Analysis

Continuous data in the descriptive patient characteristics were shown as median (interquartile range) while categorical data were presented in absolute numbers (frequency). Univariable regression with odds ratios with 95% Confidence interval (CI) was performed for baseline characteristics and serum parameters with a confirmed allergy as the outcome variable. Univariable and multivariable logistic regression were performed for both PEN‐FAST and CEPH‐FAST criteria. Sensitivity, specificity, positive predictive value (PPV), negative predictive value (NPV), and area under the receiver operating curve (AU‐ROC) for PEN‐FAST and CEPH‐FAST scores of two or fewer points were calculated with 95% CI. Statistical analysis was performed using IBM SPSS statistics version 29 and GraphPad Prism 10.

## 3 Results

### 3.1 Baseline Demographics

**FIGURE 1:**
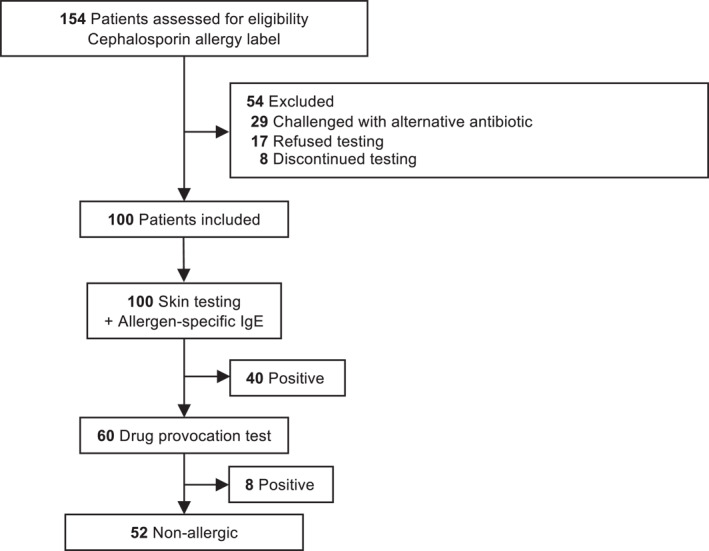
Selection of study participants and key outcomes. Data are shown as number of patients. Patients who underwent negative drug provocation tests with the implicated cephalosporin were defined as non‐allergic.

In total, 154 patients with a documented cephalosporin allergy were identified (Figure 1). However, 54 patients were not included in the final analysis, as testing with the implicated cephalosporin was not completed (Figure 1). The study population was predominantly female (74%) (Table 1). The most frequently reported cephalosporin was cefuroxime. There were no patients that carried an allergy label for two or more cephalosporins. In total, 24 patients carried an additional penicillin‐allergy label, and 39 carried additional non‐penicillin antibiotic allergy labels.

**TABLE 1:** Baseline characteristics of included patients carrying a cephalosporin allergy label.

Characteristic	Overall (*N* = 100)	Any cephalosporin test positive (*n* = 48)	PEN‐FAST score 3–5 (*n* = 80)	CEPH‐FAST score 3–5 (*n* = 58)
Sex				
Male	26 (26.0)	10 (20.8)	22 (27.5)	15 (25.9)
Female	74 (74.0)	38 (79.2)	58 (72.5)	43 (74.1)
Age, years (IQR)	50 (36–59)	51 (37–57)	50 (37–59)	48 (37–60)
Allergy phenotype				
Non‐immune‐mediated	3 (3.0)	0 (0)	2 (2.5)	1 (1.7)
Immediate immune‐mediated	44 (44.0)	25 (52.1)	44 (55.0)	38 (65.5)
Delayed immune‐mediated	32 (32.0)	16 (33.3)	22 (27.5)	13 (22.4)
Unclear/unknown	21 (21.0)	7 (14.6)	12 (15.0)	6 (10.3)
Reported allergy label				
Cefaclor	16 (16.0)	9 (18.8)	10 (12.5)	9 (15.5)
Cefadroxil	2 (2.0)	1 (2.1)	2 (2.5)	1 (1.7)
Cefazolin	7 (7.0)	2 (4.2)	7 (8.8)	6 (10.3)
Cefixime	2 (2.0)	1 (2.1)	1 (1.3)	1 (1.7)
Cefotaxime	1 (1.0)	1 (2.1)	1 (1.3)	1 (1.7)
Cefpodoxime	3 (3.0)	1 (2.1)	2 (2.5)	2 (3.4)
Ceftibuten	1 (1.0)	0 (0)	1 (1.3)	1 (1.7)
Ceftriaxone	7 (7.0)	5 (10.4)	7 (8.8)	3 (5.2)
Cefuroxime	59 (59.0)	28 (58.3)	47 (58.8)	33 (56.9)
Cephalexin	2 (2.0)	0 (0)	2 (2.5)	1 (1.7)
Concomitant antibiotic allergy labels				
Penicillin	24 (24.0)	15 (31.3)	24 (30.0)	15 (25.9)
Non‐penicillin	39 (39.0)	14 (29.2)	26 (32.5)	18 (31.0)
Reported index reaction				
Urticaria and/or rash	66 (66.0)	33 (68.8)	50 (62.5)	31 (53.4)
Angioedema	27 (27.0)	15 (31.3)	27 (33.8)	27 (46.6)
Bronchospasm/airway obstruction	19 (19.0)	11 (22.9)	19 (23.8)	18 (31.0)
Gastrointestinal symptoms	7 (7.0)	4 (8.3)	2 (2.5)	4 (6.9)
SCAR	0 (0)	0 (0)	0 (0)	0 (0)
Blistering/desquamating rash	2 (2.0)	2 (4.2)	1 (1.3)	1 (1.7)
Vasculitis	1 (1.0)	1 (2.1)	1 (1.3)	0 (0)
Hypotension	12 (12.0)	6 (12.5)	12 (15.0)	10 (17.2)
Syncope	2 (2.0)	2 (4.2)	2 (2.5)	2 (3.4)
CPR required	5 (5.0)	4 (8.3)	5 (6.3)	5 (8.6)
ICU admission required	6 (6.0)	5 (10.4)	6 (7.5)	6 (10.3)
Unclear/unknown	1 (1.0)	0 (0)	0 (0)	0 (0)
Comorbidities				
Atopy	21 (21.0)	8 (16.7)	17 (21.3)	11 (19.0)
Allergic asthma	8 (8.0)	1 (2.1)	6 (7.5)	3 (5.2)
Cardiovascular disease	19 (19.0)	8 (16.7)	16 (20.0)	10 (17.2)
Malignant disease	10 (10.0)	4 (8.3)	9 (11.3)	5 (8.6)

*Note:* Categorical data are shown as number of patients (% of patients of respective cohort). PEN‐FAST/CEPH‐FAST scores 0–2 are defined as low risk (< 5%).

Abbreviations: CPR, cardiopulmonary resuscitation; ICU, intensive care unit; SCAR, severe cutaneous adverse reaction.

Most patients (66 of 100) reported urticaria and/or rash as their index reaction, whereas angioedema and airway obstruction were present in 22% and 14%, respectively (Table 1). Cardiopulmonary resuscitation (CPR) was required for five patients, and six patients required intensive care unit (ICU) admission. Atopy was the most frequent comorbidity (21%), followed by cardiovascular disease (19%), malignant disease (10%), and allergic asthma (8%).

### 3.2 Allergy Assessment

Of the 100 included patients, 48 were confirmed as allergic, with 40 of 48 showing positive skin test results and/or elevated cefaclor‐specific IgE, and 8 of 48 patients developing an allergic reaction following DPT (Figure 1 and Table 2). There were four patients that were confirmed as allergic based on cefaclor‐specific IgE alone, while one patient showed both positive cefaclor‐specific IgE as well as a positive prick test (Table 2).

**TABLE 2:** Summary of diagnostic outcomes and clinical features from allergy assessment.

Characteristic	Overall (*N* = 100)	Any cephalosporin test positive (*n* = 48)		PEN‐FAST score 3–5 (*n* = 80)	CEPH‐FAST score 3–5 (*n* = 58)
Allergy label retained	48 (48.0)		48 (100.0)	47 (58.8)	36 (62.1)
Positive skin test	35 (35.0)		35 (72.9)	34 (42.5)	26 (44.8)
Positive prick test	16 (16.0)		16 (33.3)	16 (20.0)	16 (27.6)
Positive patch test	20 (20.0)		20 (41.7)	19 (23.8)	11 (19.0)
Positive cefaclor‐specific IgE	5 (5.0)		5 (10.4)	5 (6.3)	5 (8.6)
Positive drug provocation test	8 (8.0)		8 (16.7)	8 (10.0)	5 (8.6)
PEN‐FAST/CEPH‐FAST features					
Five years or less since reaction	86 (86.0)		46 (95.8)	77 (96.3)	56 (96.6)
Anaphylaxis/angioedema or SCAR	51 (51.0)		29 (60.4)	48 (60.0)	51 (87.9)
Systemic treatment received	48 (48.0)		32 (66.7)	46 (57.5)	46 (79.3)
Corticosteroids/AH	31 (31.0)		24 (50.0)	29 (36.3)	29 (50.0)
Epinephrine	12 (12.0)		5 (10.4)	12 (15.0)	12 (20.7)
Unspecified drug	5 (5.0)		3 (6.3)	5 (6.3)	5 (8.6)
No treatment received	11 (11.0)		1 (2.1)	2 (2.5)	2 (3.4)
Treatment category unknown	41 (41.0)		15 (31.3)	32 (40.0)	10 (17.2)
Serum markers					
Total IgE, kU/L	61.8 (28.2–149.0)		66.4 (30.6–180.0)	62.6 (29.5–155.0)	70.4 (36.2–167.0)
BTC, kU/L	4.7 (3.6–6.7)		5.1 (3.8–7.0)	4.6 (3.6–6.6)	4.6 (3.5–6.2)

*Note:* Categorical data are shown as number of patients (% of patients of respective subgroup). Continuous data are shown as median (IQR). PEN‐FAST/CEPH‐FAST scores 0–2 are defined as low risk (< 5%).

Abbreviations: AH, antihistamines; BTC, Baseline serum mast cell tryptase concentration; IV, intravenous; SCAR, severe cutaneous adverse reaction.

### 3.3 PEN‐FAST and CEPH‐FAST Scores

The majority (86%) of patients reported an index reaction in the last 5 years (Table 2). Anaphylaxis and/or angioedema were reported by 51 of 100 patients, and 48 patients reported that they had received systemic treatment. There were no patients reporting SCAR. While information was available for all patients regarding the timing of the allergic reaction, the treatment category was unknown in 41 patients (Table 2). For both PEN‐FAST and CEPH‐FAST the most frequent score was 5 points (Supporting Information S1: Table S2). Overall, 20 of 100 patients had a PEN‐FAST score of 0–2 points (=low risk), while 42 of 100 had a CEPH‐FAST score of 0–2 points.

### 3.4 Predictive Modeling of Cephalosporin Allergy Outcomes

Univariable logistic regression showed no significant association between a positive allergy outcome and baseline clinical characteristics (Table 3). In contrast, PEN‐FAST and CEPH‐FAST scores of > 2 points were significantly associated with confirmed allergic reactions (PEN‐FAST: OR 27.1, 95% CI 3.5–212.2, *p* = 0.002; CEPH‐FAST: OR 4.1, 95% CI 1.7–9.6, *p* = 0.01).

**TABLE 3:** Univariable logistic regression analysis of baseline predictors for confirmed allergies.

Clinical characteristic	Total (*N* = 100)	
	OR (95% CI)	*p*‐value
Age	1.0 (1.0–1.0)	0.81
Female sex	1.7 (0.7–4.2)	0.26
Allergy type: Immediate immune‐mediated	1.9 (0.9–4.2)	0.12
Allergy type: Delayed immune‐mediated	1.1 (0.5–2.6)	0.78
Allergy type: Unclear/unknown	0.5 (0.2–1.3)	0.14
Allergy label: Cefuroxime	1.0 (0.4–2.1)	0.90
Atopy	0.6 (0.2–1.6)	0.31
Allergic asthma	0.1 (0.0–1.2)	0.07
Cardiovascular disease	0.7 (0.2–2.1)	0.57
Malignant disease	0.8 (0.2–2.6)	0.60
Concomitant drug allergy label: Penicillin	0.8 (0.3–1.8)	0.58
Concomitant drug allergy label: Non‐penicillin antibiotic	0.4 (0.2–1.0)	0.06
Index reaction: Angioedema	1.5 (0.6–3.7)	0.36
Index reaction: Bronchospasm/airway obstruction	1.6 (0.6–4.5)	0.34
Index reaction: Gastrointestinal symptoms	1.5 (0.3–7.0)	0.62
Index reaction: Hypotension	1.1 (0.3–3.7)	0.88
Index reaction: CPR required	4.6 (0.5–43.0)	0.18
Index reaction: ICU admission required	5.9 (0.7–52.7)	0.11
Total IgE^a^	1.3 (1.0–1.8)	0.06
BTC^a^	1.4 (0.5–3.6)	0.51
PEN‐FAST score 3–5	27.1 (3.5–212.2)	**0.002**
CEPH‐FAST score 3–5	4.1 (1.7–9.6)	**0.01**

*Note:* Bold values indicate statistical significance (*p* < 0.05).

Abbreviation: BTC, Baseline serum mast cell tryptase concentration.

^a^ Tryptase and total IgE were transformed using the natural logarithm. Odds ratios represent the effect per unit increase in the log‐transformed variable.

To identify specific PEN‐FAST and CEPH‐FAST features that were associated with a positive cephalosporin allergy outcome, logistic regression analyses were performed (Table 4). A reaction occurring within 5 years or less and having received treatment for the reaction were both significant predictors in univariable analysis (*p* = 0.015 and 0.006, respectively). The associations between these features and cephalosporin allergy persisted after adjusting for all other features in the multivariable analyses (*p* = 0.011 and 0.003). Anaphylaxis and angioedema did not show a significant association in either model (*p* = 0.072 and 0.356). The “treatment unknown” category, which showed no significant association in univariable analysis (*p* = 0.110), became significant in multivariable analysis (*p* = 0.044). A supporting information table presenting the distribution of treatment categories by allergy outcome is provided (Supporting Information S1: Table S5).

**TABLE 4:** Univariable and multivariable logistic regression of PEN‐FAST and CEPH‐FAST features for confirmed allergies (any cephalosporin test positive).

Feature	Univariable analysis		Multivariable analysis	
	Odds ratio (95% CI)	*p*‐value	Odds ratio (95% CI)	*p*‐value
Five years or less	6.9 (1.5, 32.7)	**0.015**	8.6 (1.6, 44.5)	**0.011**
Anaphylaxis, angioedema or SCAR	2.1 (0.94, 4.6)	0.072	0.60 (0.21, 1.8)	0.356
No treatment received	Ref		Ref	
Treatment received	20.0 (2.4, 170.2)	**0.006**	34.6 (3.5, 347.1)	**0.003**
Treatment unknown	5.8 (0.67, 49.6)	0.110	9.6 (1.1, 86.4)	**0.044**

*Note:* Bold values indicate statistical significance (*p* < 0.05).

Abbreviations: CI, confidence interval; N/A, not applicable; SCAR, severe cutaneous adverse reaction.

### 3.5 Diagnostic Accuracy of PEN‐FAST and CEPH‐FAST

Given the more permissive scoring approach of CEPH‐FAST, particularly in patients with incomplete historical information, its diagnostic performance was analyzed in direct comparison with PEN‐FAST.

Of the 20 patients categorized as low‐risk by PEN‐FAST, one patient showed a positive patch test (Supporting Information S1: Table S3). In accordance with previous findings [14], PEN‐FAST showed a NPV of 95.0% (95% CI, 76.4, 99.7) and high sensitivity of 97.9% (95% CI, 89.1, 99.9), while PPV and specificity were lower (Table 5). AU‐ROC was 0.769 (95% CI, 0.670, 0.867) (Table 5).

**TABLE 5:** Diagnostic performance of PEN‐FAST and CEPH‐FAST in predicting confirmed allergies.

	PEN‐FAST	CEPH‐FAST
Sensitivity (95% CI)	97.9 (89.1, 99.9)	75.0 (61.2, 85.1)
Specificity (95% CI)	36.5 (24.8, 50.1)	57.7 (44.2, 70.1)
PPV (95% CI)	58.8 (47.8, 68.9)	62.1 (49.2, 73.4)
NPV (95% CI)	95.0 (76.4, 99.7)	71.4 (56.4, 82.8)
AU‐ROC (95% CI)	0.769 (0.670, 0.867)	0.667 (0.560, 0.775)

Abbreviations: AU‐ROC, area under the receiver operating curve; CI, confidence interval; NPV, negative predictive value; PPV, positive predictive value.

In contrast, CEPH‐FAST identified 42 of 100 patients suitable for direct DPT (CEPH‐FAST score 0–2). However, 12 of 42 patients showed a positive allergy outcome, with reactions distributed across different cephalosporins (Supporting Information S1: Table S3). CEPH‐FAST showed lower AU‐ROC (0.667), NPV (71.4%) and sensitivity (75.0%) than PEN‐FAST (Table 5).

A sensitivity analysis restricted to patients who completed drug provocation testing (*n* = 60) is presented in Supporting Information S1: Table S4.

## 4 Discussion

CEPH‐FAST and PEN‐FAST are both risk‐stratified approaches which aim to enable antibiotic allergy delabeling by non‐allergists without the need for skin tests. CEPH‐FAST differs from PEN‐FAST by omitting point allocation for unknown reaction timing and unknown treatment history, making it a more permissive scoring system for patients with incomplete allergy histories. Our results demonstrated that PEN‐FAST had high sensitivity and negative predictive value for cephalosporin allergies, consistent with previous validation studies of the score for predicting penicillin allergy. These findings indicate that PEN‐FAST can be successfully applied to cephalosporin allergy assessment. In contrast, CEPH‐FAST performed inferior to PEN‐FAST, with moderate sensitivity and lower negative predictive value.

A key question arising from our results is why CEPH‐FAST underperformed in our cohort compared with the findings reported by Cox et al. which reported a similar performance to that of PEN‐FAST for penicillin allergy labels [26].

It is important to underline that among patients with low CEPH‐FAST scores in our study, only a minority reacted on DPT, while the majority demonstrated positive patch tests. Hence, CEPH‐FAST was able to successfully identify patients with immediate type allergies. The distribution of reaction types was similar to Cox et al., with higher CEPH‐FAST scores corresponding to a greater likelihood of confirmed allergy [26].

Patch testing for cephalosporins is not fully standardized and its diagnostic accuracy, particularly for delayed hypersensitivity reactions, remains uncertain. This diagnostic uncertainty further limits interpretation of positive predictive values derived from skin testing alone. While a positive patch test may support the diagnosis in the context of a compatible clinical history, patch test positivity alone does not necessarily equal to clinically relevant allergy. In the present study, patch test results were interpreted in conjunction with the reported reaction phenotype and other diagnostic findings, and drug provocation testing was performed whenever patch testing was negative and clinically appropriate. Nevertheless, reliance on patch testing may have contributed to an overestimation of confirmed allergy in a subset of patients, which should be considered when interpreting the performance of CEPH‐FAST.

In an additional analysis restricted to patients who underwent DPT, PEN‐FAST retained a high negative predictive value (100%), while CEPH‐FAST demonstrated a lower negative predictive value (90.1%) than PEN‐FAST but higher than in the primary analysis including skin testing (71.4%). This finding suggests that inclusion of skin test‐based outcomes may influence the observed performance of the clinical scores and further underscores the need for prospective studies incorporating direct drug challenges.

It can be speculated that the proportion of patients with unknown treatment histories might lead to the observed discrepancy in the performance of CEPH‐FAST. Nevertheless, the cohorts described by Cox et al. exhibited a large variability of the proportion of patients with unknown treatment histories from 15.4% to 73.7%. Since our study offers a comparable patient cohort with 41%, this factor alone is unlikely to explain the observed differences in CEPH‐FAST performance.

Another factor which may contribute to the observed discrepancy is the variation in analyzed cephalosporin agents. Accordingly, the most frequent allergy label in the previously published study, performed in Australia and North America, was cephalexin, which constituted only a very small minority in our study. In many European countries, cefuroxime is the most frequently administered cephalosporin [4] and was subsequently the most common allergy label in our study, while only rarely reported by Cox et al. [26].

Despite the lower performance of CEPH‐FAST observed in this study, the findings highlight the value of structured clinical decision rules in the assessment of reported cephalosporin allergy.

We examined baseline clinical variables, including details of the index reaction and patient characteristics to explore associations with confirmed allergies. Our results demonstrated that the analyzed clinical variables were not significantly associated with a confirmed allergy. In contrast, both the PEN‐FAST and CEPH‐FAST scores emerged as significant predictors, indicating that structured clinical decision rules provide a far more accurate assessment of true allergy risk than evaluating patients based on individual symptoms alone. Several other clinical pathways and prediction tools have been proposed to support penicillin allergy risk stratification and delabeling, including structured clinical pathways such as the APAAACI Clinical Pathway and the more recent BL‐predictor [29, 30]. While these approaches share the aim of identifying patients at low risk by non‐allergy specialists who may safely undergo direct drug challenge, they differ in complexity, required clinical input, and validation populations. Direct comparative data between these tools and simplified scoring systems such as PEN‐FAST and CEPH‐FAST remain limited. Our findings support ongoing efforts to implement such tools on cephalosporins to enable broader, evidence‐based antibiotic allergy delabeling.

The diagnostic performance of clinical decision rules such as PEN‐FAST and CEPH‐FAST must be interpreted in the context of the diagnostic pathway used to define allergy outcomes. Skin testing and serum specific IgE remain less well validated for cephalosporins than for penicillin and do not constitute definitive reference standards in the absence of systematic drug provocation testing. Interpretation of predictive values in this setting requires particular caution. For clinical decision rules designed to identify patients suitable for direct drug provocation testing, negative predictive value represents the most clinically relevant measure of safety. In contrast, positive predictive value is highly dependent on the reference standard used to define allergy status and is therefore constrained in retrospective cohorts where drug provocation testing is not performed systematically. As a result, the apparent positive predictive value of skin testing and serum specific IgE for cephalosporins is likely to be overestimated. In our cohort, the combined NPV of specific IgE and skin testing was higher than that of CEPH‐FAST but remained below the level observed for PEN‐FAST. Given the potential for false‐positive skin test results among patients classified as low risk by CEPH‐FAST (0–2 points), the true predictive performance of this clinical rule may in fact be higher than observed, warranting further prospective validation. The diagnostic value of specific IgE also remains controversial [31]. Although its sensitivity is limited and it should be interpreted alongside skin test results [32], previous studies have shown high positive predictive value of elevated cefaclor‐specific IgE [33]. In the present study, a small number of patients were classified as having confirmed allergy on the basis of isolated serum specific IgE positivity, which may overestimate true allergy. However, this applied to four patients only, all of whom had high‐risk clinical histories and PEN‐FAST or CEPH‐FAST scores of 4 or 5.

Clinical decision rules such as PEN‐FAST and CEPH‐FAST are intended to support antibiotic allergy risk stratification and delabeling by non‐specialists. This study does not evaluate the use of CEPH‐FAST in non‐specialist settings as the score was applied retrospectively to allergy histories obtained within a specialist clinic. Accordingly, these findings should be interpreted as reflective of the intrinsic performance of the scoring system in a specialist clinic setting, rather than as definitive evidence to support implementation in non‐specialist delabeling pathways. Prospective evaluation in non‐specialist settings is required before broader use can be recommended.

Limitations of our study are retrospective design and referral bias with a high percentage of confirmed allergies. Nevertheless, the overall rate of confirmed allergy in our study aligned closely with findings from previous investigations [26]. Our findings may not be applicable to other institutions with differing patient demographics and clinical practices. As the study was conducted in a tertiary referral setting, the findings may not fully reflect the performance of these decision rules in primary care or peri‐operative contexts, where cephalosporin allergy labels are particularly relevant. The relatively small sample size limits statistical power, particularly for subgroup analyses, and may have contributed to variability in the observed predictive performance of the clinical scores. Nevertheless, the performance of PEN‐FAST was similar to earlier studies with larger sample sizes. In addition, the retrospective design limited standardized documentation of allergy histories and application of diagnostic procedures. The “treatment unknown” category may represent heterogeneous scenarios, including patients who received treatment but were unable to recall the specific medication, as well as patients who could not recall whether any treatment was administered. This distinction could not be reliably determined due to the retrospective study design and may have influenced the observed associations in the regression analysis. Cephalosporin skin testing and specific IgE assays are not fully standardized, and variability in methods and interpretation may further limit comparability with other studies. Intradermal testing (IDT) was not performed. This may have influenced diagnostic classification in a subset of patients, as IDT can increase sensitivity in some immediate reactions but may also introduce false‐positive results depending on protocol and interpretation. Although negative skin tests were followed by drug challenges, the absence of IDT contributes to diagnostic uncertainty and may have affected observed test and score performance. Furthermore, our study population did not include patients with SCAR and thus the results cannot be applied to this subgroup with high‐risk features that are not suitable for delabeling by non‐allergists.

## 5 Conclusions

PEN‐FAST demonstrated high sensitivity and negative predictive value for identifying low‐risk cephalosporin allergy and may support clinical risk stratification for antibiotic allergy delabeling. In contrast, CEPH‐FAST showed reduced diagnostic performance in this cohort and requires further validation before routine clinical use. These findings suggest that structured clinical decision rules may contribute to improved assessment of reported cephalosporin allergy.

## Author Contributions

**Deniz Göcebe:** conceptualization, investigation, writing – original draft, writing – review and editing, visualization, validation, methodology, formal analysis, data curation. **Hannah Nürnberg:** writing – review and editing, conceptualization. **Alexander Enk:** conceptualization, writing – review and editing, supervision, methodology. **Elham Khatamzas:** conceptualization, writing – review and editing, supervision. **Knut Schäkel:** conceptualization, writing – review and editing, supervision, validation, methodology.

## Ethics Statement

The study was performed according to the Declaration of Helsinki and approved by the local ethics committee of the medical faculty of the University of Heidelberg, Germany (S‐004/2025). Data collection did not require informed consent. Data were obtained after approval of the ethics committee.

## Conflicts of Interest

The authors declare no conflicts of interest.

## Supporting information


Supporting Information S1

## Data Availability

The data that support the findings of this study are available on request from the corresponding author. The data are not publicly available due to privacy or ethical restrictions.
