# Supplementary material for: Performance of PEN‐FAST and CEPH‐FAST for Cephalosporin Allergy Delabeling
Source: Clin Transl Allergy. 2026 May 27;16(6):e70178. doi: 10.1002/clt2.70178 (PMC13239963; doi:10.1002/clt2.70178)
Supplement: Supplementary file 1 — Supporting Information S1 [file CLT2-16-e70178-s001.docx]

**Supplementary Data**

**Inclusion criteria**

1. Adult patients with a cephalosporin allergy label who were referred to the Allergy Department at the University Hospital of Heidelberg, Germany, tested between 2004 and 2024. Eligible patients had either their allergy confirmed by a positive result in skin testing or cephalosporin-specific IgE measurement or had undergone a drug provocation test with the cephalosporin they were suspected to be allergic to.

**Exclusion criteria**

1. Patients under 18 years of age.
2. Patients who declined to undergo skin testing, specific IgE testing, or a drug provocation test.
3. Cases in which allergy testing was discontinued prior to completion.
4. Drug challenge performed with a cephalosporin different from the one suspected of causing the reaction.
5. Patients who did not undergo drug challenge despite negative skin test results and/or negative specific IgE.
6. Current treatment with steroids equivalent to ≥20 mg prednisolone per day.
7. Ongoing antihistamine therapy.
8. Pregnancy.
9. Skin conditions present at the skin testing site.
10. Markedly reduced general health status.
11. Bronchial asthma that was unstable or inadequately controlled.
12. History of stem cell transplantation.
13. Previous diagnosis of acute interstitial nephritis.
14. Chronic urticaria.
15. Diagnosis of mastocytosis.

**Patient identification**

Patients were identified via the hospital’s electronic patient management system by analyzing the relevant ICD-coded worklists from the Allergy Department. Each case was then manually reviewed in full.

**PEN-FAST and CEPH-FAST score determination**

PEN-FAST scores (ranging from 0-5) were calculated in accordance with previously published criteria.^1^

- 2 points were assigned if the reaction occurred within the last five years or if the timing was unknown.
- 2 points were assigned for anaphylaxis, angioedema, or severe cutaneous adverse reactions (SCAR).
- 1 point was assigned if hospitalization or systemic treatment was required, or if this information was unknown.

CEPH-FAST scoring followed previously established criteria as well.^2^ In contrast to PEN-FAST, CEPH-FAST does **not** assign points if the reaction timing or treatment details are unknown.

**Definition of anaphylaxis**

Anaphylaxis was defined per the original PEN-FAST study by Trubiano et al. as a cutaneous reaction accompanied by at least one of the following: respiratory, cardiovascular, or gastrointestinal symptoms.^1^ It was also diagnosed if hypotension, bronchospasm, or airway obstruction occurred without skin involvement.

**Severe cutaneous adverse reactions**

As per the original PEN-FAST study, SCARs included Stevens-Johnson Syndrome (SJS), Toxic Epidermal Necrolysis (TEN), Acute Generalized Exanthematous Pustulosis (AGEP), and Drug Reaction with Eosinophilia and Systemic Symptoms (DRESS).^1^ Patients presenting with skin symptoms alongside mucosal ulceration also received two points.^1^

**Skin testing**

Skin prick testing was performed using a concentration of 20 ng/mL. Prick tests were evaluated after 20 minutes; a wheal of ≥2 mm compared to the negative control was considered positive. A 0.9% sodium chloride solution served as the negative control. Histamine dihydrochloride (1 mg/mL, Allergopharma, Germany) was used as the positive control for skin prick tests.

Intradermal tests were not performed as the hospital’s local protocol does not include them in standard practice.

Patch tests were carried out using Finn Chambers (SmartPractice, Germany). Patch tests were conducted using a 5% aqueous solution. Readings were taken at 48 and 72 hours. 0.9% sodium chloride and 0.25% sodium lauryl sulphate were used as negative and positive controls, respectively. Patch test reactions were graded according to local protocol, with erythema and infiltration (+) representing the minimum criterion for a positive result.

Patients with a positive prick and/or patch test for the implicated cephalosporin were confirmed as allergic.

**Serum markers**

As part of the routine diagnostic evaluation, total serum IgE and serum tryptase levels were measured. Levels of total and cephalosporin-specific IgE (only available for cefaclor) were assessed using fluorescent enzyme immunoassays (ImmunoCAP, Thermo Fisher).

**Drug provocation test**

All drug provocation tests were performed using the culprit cephalosporin. A 2-step oral challenge (50%-50%) with a therapeutic dose was performed with an interval of 30 minutes. If the culprit cephalosporin was not available as an oral drug, an intravenous challenge with a full therapeutic dose was performed, following the hospital’s drug challenge protocol. Following drug provocation testing, patients were observed for approximately four hours in the outpatient setting. In cases considered higher risk, observation was extended and patients were monitored overnight in the inpatient setting.

**Supplementary Table S1.** PEN-FAST and CEPH-FAST scores.

| **Criteria** | | **Point score** | |
| --- | --- | --- | --- |
|  |  | **PEN-FAST** | **CEPH-FAST** |
| Reaction occurred in last five years | |  |  |
|  | Yes | 2 | 2 |
|  | No | 0 | 0 |
|  | Unknown | 2 | 0 |
| Anaphylaxis, angioedema or SCAR | |  |  |
|  | Yes | 2 | 2 |
|  | No | 0 | 0 |
|  | Unknown | 0 | 0 |
| Treatment received | |  |  |
|  | Yes | 1 | 1 |
|  | No | 0 | 0 |
|  | Unknown | 1 | 0 |

**Abbreviations:** SCAR, severe cutaneous adverse reaction.

**Supplementary Table S2.** PEN-FAST and CEPH-FAST score distributions.

|  | | **Overall**  **(N=100)** | **Confirmed allergy**  **(n=48)** | **Non-allergic**  **(n=52)** |
| --- | --- | --- | --- | --- |
| PEN-FAST scores | |  |  |  |
|  | PEN-FAST 0 | 0 (0) | 0 (0) | 0 (0) |
|  | PEN-FAST 1 | 11 (11.0) | 0 (0) | 11 (21.2) |
|  | PEN-FAST 2 | 9 (9.0) | 1 (2.1) | 8 (15.4) |
|  | PEN-FAST 3 | 34 (34.0) | 19 (39.6) | 15 (28.8) |
|  | PEN-FAST 4 | 1 (1.0) | 1 (2.1) | 0 (0) |
|  | PEN-FAST 5 | 45 (45.0) | 27 (56.3) | 18 (34.6) |
| CEPH-FAST scores | |  |  |  |
|  | CEPH-FAST 0 | 9 (9.0) | 0 (0) | 9 (17.3) |
|  | CEPH-FAST 1 | 2 (2.0) | 0 (0) | 2 (3.8) |
|  | CEPH-FAST 2 | 31 (31.0) | 12 (25.0) | 19 (36.5) |
|  | CEPH-FAST 3 | 12 (12.0) | 8 (16.7) | 4 (7.7) |
|  | CEPH-FAST 4 | 11 (11.0) | 5 (10.4) | 6 (11.5) |
|  | CEPH-FAST 5 | 35 (35.0) | 23 (47.9) | 12 (23.1) |

Data are shown as number of patients (% of patients of the respective cohort).

**Supplementary Table S3.** Characteristics of patients incorrectly identified by PEN-FAST and/or CEPH-FAST.

| **Patient** | 1 | 2 | 3 | 4 | 5 | 6 |
| --- | --- | --- | --- | --- | --- | --- |
| **Incorrectly identified by** | PEN-FAST, CEPH-FAST | CEPH-FAST | CEPH-FAST | CEPH-FAST | CEPH-FAST | CEPH-FAST |
| **Sex** | Female | Female | Male | Female | Male | Female |
| **Age** | 31 | 23 | 53 | 70 | 78 | 50 |
| **Reported allergy label** | Cefuroxime | Cefuroxime | Cefuroxime | Cefuroxime | Cefuroxime | Cefuroxime |
| **Reported index reaction** | Desquamating rash^†^ | Urticaria/rash | Urticaria/rash | Urticaria/rash | Urticaria/rash, vasculitis^‡^ | Urticaria/rash |
| **Reported onset latency** | 1 day | Unclear/unknown | 2 days | Unclear/unknown | Several days | 2 days |
| **Allergy type** | Delayed | Unclear/unknown | Delayed | Unclear/unknown | Unclear/unknown | Delayed |
| **PEN-FAST score** | 2 | 3 | 3 | 3 | 3 | 3 |
| **CEPH-FAST score** | 2 | 2 | 2 | 2 | 2 | 2 |
| **Five years or less since reaction** | Yes | Yes | Yes | Yes | Yes | Yes |
| **Anaphylaxis/Angioedema or SCAR** | No | No | No | No | No | No |
| **Treatment required for reaction** | No | Unclear/unknown | Unclear/unknown | Unclear/unknown | Unclear/unknown | Unclear/unknown |
| **Allergen-specific IgE (kU/l)** | N/A | N/A | N/A | N/A | N/A | N/A |
| **Prick test** | Negative | Negative | Negative | Negative | Negative | Negative |
| **Patch test** | Positive (Cefuroxime) | Negative | Positive (Cefuroxime) | Positive  (Cefuroxime) | Positive  (Cefuroxime) | Positive  (Cefuroxime) |
| **Drug provocation test result*** | Not performed | Positive (rash) | Not performed | Not performed | Not performed | Not performed |

**Abbreviations:** N/A, not applicable; SCAR, severe cutaneous adverse reaction.

^†^Palmoplantar desquamation approximately one week after onset, no mucosal involvement.

^‡^Clinically diagnosed leukocytoclastic vasculitis of the legs after several days.

**Supplementary Table S3.** continued.

| **Patient** | 7 | 8 | 9 | 10 | 11 | 12 |
| --- | --- | --- | --- | --- | --- | --- |
| **Incorrectly identified by** | CEPH-FAST | CEPH-FAST | CEPH-FAST | CEPH-FAST | CEPH-FAST | CEPH-FAST |
| **Sex** | Female | Female | Female | Female | Female | Female |
| **Age** | 57 | 59 | 54 | 55 | 51 | 55 |
| **Reported allergy label** | Cefuroxime | Cefadroxil | Cefuroxime | Cefaclor | Ceftriaxone | Ceftriaxone |
| **Reported index reaction** | Urticaria/rash | Urticaria/rash | Urticaria/rash | Urticaria/rash | Urticaria/rash | Urticaria/rash |
| **Reported onset latency** | 4 days | 2 hours | Unclear/unknown | 3 days | Unclear/unknown | 3-4 days |
| **Allergy type** | Delayed | Immediate | Unclear/unknown | Delayed | Unclear/unknown | Delayed |
| **PEN-FAST score** | 3 | 3 | 3 | 3 | 3 | 3 |
| **CEPH-FAST score** | 2 | 2 | 2 | 2 | 2 | 2 |
| **Five years or less since reaction** | Yes | Yes | Yes | Yes | Yes | Yes |
| **Anaphylaxis/Angioedema or SCAR** | No | No | No | No | No | No |
| **Treatment required for reaction** | No | Unclear/unknown | Unclear/unknown | Unclear/unknown | Unclear/unknown | Unclear/unknown |
| **Allergen-specific IgE (kU/l)** | N/A | N/A | N/A | Not performed | N/A | N/A |
| **Prick test** | Negative | Negative | Negative | Negative | Negative | Negative |
| **Patch test** | Positive  (Cefuroxime) | Negative | Positive  (Cefuroxime) | Positive (Cefaclor) | Negative | Positive  (Ceftriaxone) |
| **Drug provocation test result** | Not performed | Positive (erythema) | Not performed | Not performed | Positive (nausea and circulatory symptoms)^§^ | Not performed |

**Abbreviations:** N/A, not applicable; SCAR, severe cutaneous adverse reaction.

^§^No drug treatment required.

**Supplementary Table S4.** Diagnostic performance of PEN-FAST and CEPH-FAST for patients receiving drug provocation testing.

|  | **PEN-FAST** | **CEPH-FAST** |
| --- | --- | --- |
| Sensitivity (95% CI) | 100.0 (67.6,100.0) | 62.5 (30.6, 86.3) |
| Specificity (95% CI) | 36.5 (24.8, 50.1) | 57.7 (44.2, 70.1) |
| PPV (95% CI) | 19.5 (10.2, 34.0) | 18.5 (8.2, 36.7) |
| NPV (95% CI) | 100.0 (83.2, 100.0) | 90.1 (76.4, 96.9) |
| AU-ROC (95% CI) | 0.402 (0.257, 0.548) | 0.547 (0.399, 0.696) |

**Abbreviations:** AU-ROC, area under the receiver operating curve; CI, confidence interval; NPV, negative predictive value; PPV, positive predictive value.

**Supplementary Table S5.** Distribution of treatment categories by allergy outcome.

| **Treatment category** | **Total**  **(N=100)** | **Confirmed allergy**  **(n=48)** | **Non-allergic**  **(n=52)** |
| --- | --- | --- | --- |
| No treatment | 11 (11.0) | 1 (2.1) | 10 (19.2) |
| Treatment received | 48 (48.0) | 32 (66.7) | 16 (30.8) |
| Treatment unknown | 41 (41.0) | 15 (31.3) | 26 (50.0) |

Data are shown as number of patients (% of patients of the respective cohort).

**References**

1. Trubiano JA, Vogrin S, Chua KYL, et al. Development and Validation of a Penicillin Allergy Clinical Decision Rule. *JAMA Intern Med*. May 1 2020;180(5):745-752. doi:10.1001/jamainternmed.2020.0403

2. Cox F, Vogrin S, Sullivan RP, et al. Development and Validation of a Cephalosporin Allergy Clinical Decision Rule. *J Infect*. Apr 25 2025:106495. doi:10.1016/j.jinf.2025.106495
